# Viral Network Analyzer (VirNA): A Novel Minimum Spanning Networks Algorithm for Investigating Viral Evolution

**DOI:** 10.3390/ijms26052008

**Published:** 2025-02-25

**Authors:** Giorgia Mazzotti, Luca Bianco, Enrico Lavezzo, Martina Bado, Stefano Toppo, Paolo Fontana

**Affiliations:** 1 Department of Molecular Medicine, University of Padua, 35131 Padua, Italy; giorgia.mazzotti@studenti.unipd.it (G.M.); enrico.lavezzo@unipd.it (E.L.); martina.bado@studenti.unipd.it (M.B.); 2 Edmund Mach, 38098 Trento, Italy; luca.bianco@fmach.it

**Keywords:** viruses, evolution, minimum spanning networks

## Abstract

Next Generation Sequencing technologies are essential in public health surveillance for tracking pathogen evolution, spread, and the emergence of new variants. However, the extensive sequencing of viral genomes during recent pandemics has highlighted the limitations of traditional molecular phylogenetic algorithms in capturing fine-grained evolutionary details when analyzed sequences are highly similar and datasets are large-scale. VirNA (Viral Network Analyzer) addresses this challenge by reconstructing detailed mutation patterns and tracing pathogen evolutionary routes in specific geographical regions through Minimum Spanning Networks. It enables users to analyze thousands of sequences, generating networks where nodes represent genomic sequences linked to their metadata, while edges represent potential evolutionary pathways. VirNA is a powerful tool for analyzing large, high-quality datasets, providing detailed insights into rapid pathogen evolution over short time periods, with potential applications in pandemic surveillance.

## 1. Introduction

The integration of Next Generation Sequencing technologies into pandemic surveillance has generated vast amounts of data, posing challenges for sequence management and interpretation. These data are essential for tracking emerging variants and reconstructing viral transmission routes. Recent global threats like SARS-CoV-2 and Human Monkeypox Virus have accelerated whole genome sequencing, generating large genomic datasets and reshaping our understanding of viral evolution.

Molecular phylogeny is widely used to study pathogen evolution and performs best when dealing with limited amounts of data and long-term evolutionary relationships. Nonetheless, traditional phylogenetic algorithms often struggle with data from rapidly evolving pathogens and short time scales, producing low-confidence trees due to the high similarity of viral sequences [1]. To overcome this limitation, we developed VirNA (Viral Network Analyzer, v. 1.0), a tool that leverages Minimum Spanning Networks (MSNs) to reconstruct potential evolutionary trajectories of rapidly evolving viral genomes. This approach is specifically designed to accommodate the short timescales and high sequence similarities characteristic of such genomic datasets. In contrast to phylogenetic analyses, which represent observed sequences as leaves (Operational Taxonomic Units, OTUs) and internal nodes as inferred hypothetical ancestors within a hierarchical structure, MSNs directly represent relationships among observed sequences, with all nodes corresponding to actual data. Moreover, MSNs are networks, not hierarchical trees, allowing them to include loops or multiple paths in their topology to capture the complexity of evolutionary relationships. VirNA sets itself apart from existing MSN tools, like Pegas [2] and PopArt [3], by using a new way to connect sequences and enabling directional links between them. This makes it possible to trace possible routes followed by the infection. As a result, the networks can include closed paths that, because of their directional flow, are not loops. Moreover, VirNA has been implemented to efficiently analyze thousands of viral genomes. To validate the performance of VirNA, we compared the results of VirNA to those obtained from PopArt [3] and Pegas [2], highlighting their key algorithmic differences. Additionally, we applied VirNA to datasets of real viral sequences and compared its output to that of phylogeny algorithms, showing their complementary nature.

## 2. Results

### 2.1. VirNA

VirNA is an implementation of Bandelt et al.’s [4] algorithm with the introduction of novel elements for the analysis of viral genome evolution. In Bandelt’s algorithm [4], the MSN is constructed by connecting sequences based on pairwise distances, prioritizing the smallest possible differences [4]. The network is constructed through recursive steps, starting from the unconnected nodes [4]. Pairwise distances between nodes are then considered in ascending order, adding connections until the network becomes fully connected [4]. This algorithm is especially suitable for analyzing closely related sequences, such as viral genomes or population-level genetic data [4]. The final network reconstructed by Bandelt et al.’s algorithm is undirected and unrooted [4]. Consistently with the original Bandelt’s algorithm [4], VirNA traces the shortest paths that connect the sequences/nodes, but with the addition of a constraint: the set of genomic mutations in a parent node must be fully contained within those of its child node. This feature starts from the assumption that mutations tend to occur and accumulate in a forward progression starting from a reference/ancestor sequence rather than reverting to their original state. For example, when an adenine base mutates into a thymine (initiating the creation of a variant), the probability of it reverting back to adenine through subsequent mutations in the evolutionary lineage is relatively low, particularly over short evolutionary timescales and under stable environmental conditions. While such reversions may occur over extended periods due to random chance, environmental pressure, etc., they are statistically improbable in the context of short-term evolution, where the accumulation of mutations tends to dominate. From this point forward, we will refer to this new constraint introduced in our MSN implementation as Haplotype Compatibility (HC). The edges between nodes are directed and represent the accumulation of new mutations in the child node, preserving the history of accumulated mutations from the ancestor or reference sequence. As a result, the algorithm forms a Directed Acyclic Graph (DAG), with edges directed from source nodes (fewer mutations) to target nodes (more mutations), adding a novel element compared to Bandelt’s algorithm [4]. Additionally, unlike Bandelt’s algorithm [4], VirNA may split data into multiple distinct networks due to the mutation compatibility constraint.

**Figure 1:**
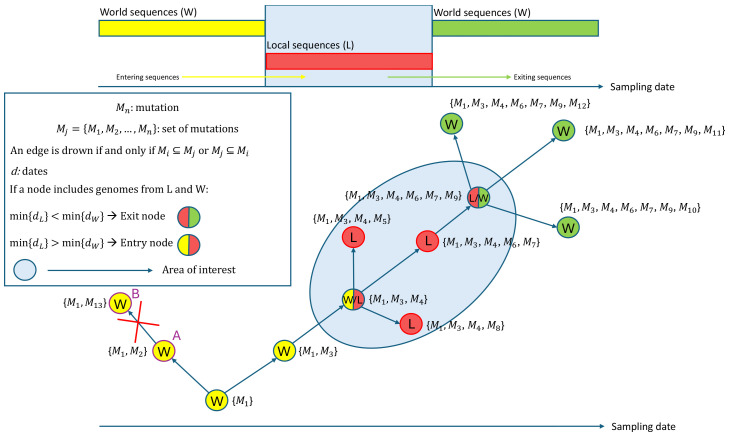
The figure depicts how VirNA algorithm works on a hypothetical example where twelve different viral genomic sequences are available. A connection between two nodes is established if and only if the mutations that define the target node precisely correspond to those characterizing the source node, complemented by additional mutations. VirNA is able to identify potential “entry” and “exit” nodes in the network. When a node in the network clusters both “Local” and “World” sequences (double colored nodes), if the earliest sampling date among the “Local” sequences is earlier than the earliest sampling date among the “World” sequences, the node is labeled as “exit” node (red and green node), conversely as “entry” node (red and yellow node).

The conceptual framework of VirNA is shown in Figure 1. In this network, nodes represent identical genomic sequences while directed edges represent potential evolutionary relationships based on the mutation accumulation principle. Node A (mutation set {m1, m2}) is not connected to node B (mutation set {m1, m13}), due to the absence of mutation m2 in B, which breaks the compatibility requirement. While this can pose a constraint in the algorithm, availability of sequence-associated metadata can enable the user to eventually draw a new connection.

VirNA also identifies potential viral introductions or exits from specific geographical areas. We define the local area as “L” (“L” for “Local”, Figure 1) and all other regions as “W” (“W” for “World”, Figure 1). “Entry” nodes mark pathogen imports from outside the local area, while “exit” nodes indicate potential spread to other regions. If the earliest “Local” sequence predates the earliest “World” sequence in a mixed node, it is labeled as an “exit” node; otherwise, it is labeled as an “entry” node. Figure 1 illustrates an example: double-colored nodes represent sequences from both “Local” and “World” regions. The timeline indicates that local sequences were sampled in the middle period, with global sequences sampled earlier or later. The red-yellow node represents an entry point where local sequences followed world sequences, while the green-yellow node is an exit point with local sequences sampled earlier.

**Figure 2:**
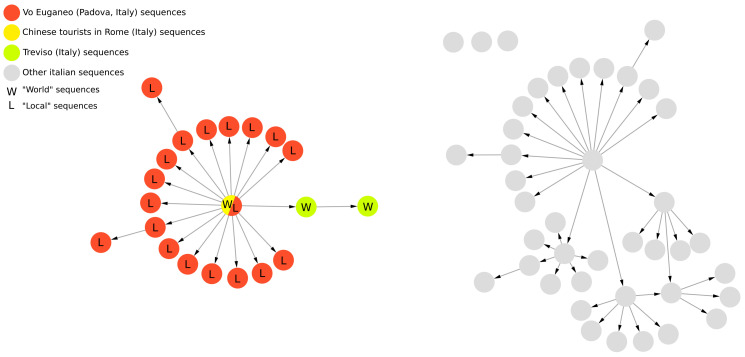
Applicative example of geographical analysis feature of VirNA on a group of SARS-CoV-2 sequences sampled in Italy from January to March 2020 and retrieved from GISAID database [5].

This feature was applied on a controlled set of real data comprising all the sequences sampled in the Italian municipality of Vo’ (Padua, Italy) between February and March of 2020, when the town was quarantined due to the first COVID-19-related deaths in Italy. The dataset, which is available on the GitHub page of VirNA (https://github.com/font71/VirNA, accessed on 20 February 2025), comprises a set of SARS-CoV-2 sequences downloaded from the GISAID database [5] and sampled between January and March 2020 in Italy. The sequences are all associated with complete collection dates (“YYYY-MM-DD” format) and locations (“Continent/Country/City” format). None of the sequences includes ambiguous characters. The geographical analysis efficiently highlights the “entry” in Vo’ of the haplotype carried by a couple of Chinese tourists that traveled in Italy in January 2020, as previously observed in Manuto et al. [6]. The spread of this haplotype was mostly contained by the lockdown measure adopted. In the same time span descendants of the tourists’ haplotype were detected also in the near city of Treviso (Italy). This could represent an “exit” event from Vo’ (as detected by VirNA because of the choice of Vo’ sequences as “Local” sequences) or another “entry” event from Rome in Treviso. These details can be observed in Figure 2 where the yellow and red node represents both the tourists (“W” sequences) and Vo’ sequences (“L” sequences), the red nodes represent Vo’ sequences, while the green nodes represent sequences sampled in Treviso.

### 2.2. Comparison with State-of-the Art Tools

**Figure 3:**
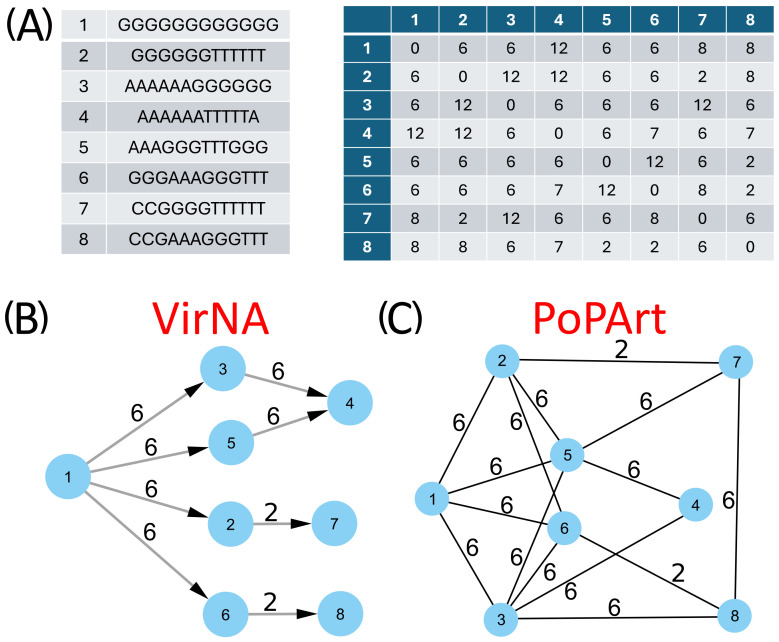
VirNA and Bandelt’s original approach, here represented by PoPArt output, are applied to the same set of eight sequences (panel (**A**)), alongside their corresponding distance matrix. The resulting Minimum Spanning Networks are displayed in panels (**B**,**C**), illustrating the different network topologies produced by VirNA and PoPArt [3], respectively.

The comparative analysis of the networks generated by PoPArt [3], Pegas [2], and VirNA, confirms distinct topological structures arising from the different algorithms implemented by the tools. Figure 3 provides a straightforward example of the final output derived from eight simple sequences, comparing the results of VirNA with those from Bandelt’s algorithm [4] as implemented in PoPArt [3]. The observations made for PoPArt [3] are also applicable to Pegas [2].

The PoPArt [3] network represents the union of all possible minimum spanning trees connecting the nodes (Figure 3, panel C). In contrast, VirNA produces a unique directed path (Figure 3, panel B). For example, VirNA does not link sequence 5 to sequence 3, unlike PoPArt [3], because this requires three reversing mutations that break the Haplotype Compatibility rule in our method.

VirNA generates a DAG where the progression of evolution is explicitly represented. Starting from a defined reference sequence, VirNA traces the accumulation of mutations over time, creating edges that connect an ancestral node to its descendants while respecting the minimum spanning differences. This approach ensures that the network has a clear origin and direction, aligning with the biological concept of evolutionary progression. In contrast, both PoPArt [3] and Pegas [2] produce cyclic graphs, as they do not assume a reference sequence or impose a direction of evolution. Instead, these tools generate connections based on minimal pairwise distances between sequences, resulting in undirected networks. This lack of an evolutionary direction means that the networks reconstructed by PoPArt [3] and Pegas [2] do not explicitly reflect the temporal progression of mutations. Consequently, while these tools provide a comprehensive representation of genetic relationships, they may mask the evolutionary dynamics of the virus.

The directed nature of VirNA networks offers several advantages in scenarios where tracking the progression of mutations is critical. For instance, VirNA is particularly effective for studying closely related sequences in confined temporal and spatial contexts, such as localized outbreaks or contact tracing studies. The Haplotype Compatibility rule ensures that only biologically plausible connections are retained, avoiding the forced linkage of genomically distant sequences. By isolating groups of sequences that violate this rule, VirNA provides a more realistic and temporally accurate depiction of viral evolution. However, the strict assumptions underlying VirNA’s approach may lead to disconnected networks in cases where reversion events or other evolutionary complexities are significant. In contrast, the cyclic graphs produced by PoPArt [3] and Pegas [2] generate all plausible evolutionary pathways, including reversions, producing a single, fully connected network. This approach links even distant genomic sequences, providing a comprehensive view of genetic relationships. However, the absence of a defined evolutionary direction in these networks can make it challenging to infer temporal progression or distinguish ancestral nodes from descendants. The choice of tool depends on the specific goals of the analysis and the characteristics of the dataset. VirNA is preferable when the objective is to reconstruct the evolutionary trajectory of the virus, emphasizing directionality and the accumulation of mutations over time. In contrast, PoPArt [3] and Pegas [2] are more appropriate for studies focused on providing a comprehensive representation of genetic relationships, prioritizing the identification of all possible evolutionary pathways over delineating a clear direction of evolution, even if this involves merging distinct haplotypes that lack shared evolutionary traits.

**Figure 4:**
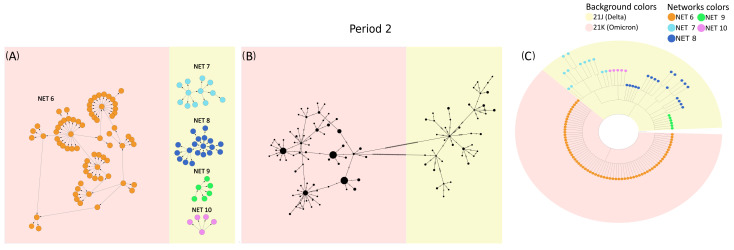
Sequences from time period 2 of the SARS-CoV-2 case study: networks generated by VirNA (panel (**A**)), network computed by PoPArt [3] (panel (**B**)), and phylogenetic tree constructed using RAxML [7] (panel (**C**)). In the MSNs computed by VirNA (panel (**A**)), the direction of arrows follows the principle of mutation accumulation. The background color in the phylogenetic tree (panel (**C**)) represents the viral variants of the terminal nodes and is consistently applied to the networks in panels (**A**,**B**). The color coding of networks in VirNA MSNs (panel (**A**)) is mirrored in the terminal nodes of the phylogenetic tree (panel (**C**)).

To further investigate these differences, VirNA, PoPArt [3], and Pegas [2] were applied to the set of SARS-CoV-2 sequences sampled from 18 December 2021 to 22 December 2021 (time period 2 of the dataset described in the Supplementary Materials). Panel A of Figure 4 demonstrates how VirNA effectively separates genomically distinct variants, such as Omicron (highlighted with a pink background) and Delta (highlighted with a light yellow background). Similarly, the phylogenetic tree in panel C clearly shows the distinct origins of these variants. In contrast, PoPArt [3], due to its algorithmic requirement to construct a single hyperconnected network, links genomically distant sequences. This is evident in panel B, where Omicron (pink background) sequences are connected to Delta (light yellow background) sequences through two long branches. Moreover, while VirNA identifies genomic subgroups within the Delta variant, PoPArt [3] merges all sequences into a single network. The approach of PoPArt [3] represents an alternative in cases like this one, where the sequence data are potentially compatible with reversions. The distinct methodologies used by VirNA and PoPArt [3] are evident: VirNA generates networks that reflect a directed evolutionary history obtained using a starting constraint, whereas PoPArt [3] produces a single comprehensive network encompassing all possible evolutionary pathways without assuming any specific evolutionary flow. The observations regarding PoPArt [3] are equally applicable to Pegas [2].

### 2.3. Comparison with Phylogeny Results

The comparison between the output topologies generated by MSNs and phylogenetic approaches in two analyzed case studies highlights their strongly complementary nature. VirNA successfully elucidated evolutionary relationships among closely related sequences in both a SARS-CoV-2 sequences and a Monkeypox sequences datasets. VirNA was particularly effective in identifying and distinguishing co-circulating viral haplotypes through the reconstruction of networks of sequences that adhered to the algorithm constraint: the progressive accumulation of mutations (as described earlier). VirNA produced multiple separate networks, revealing groups of sequences that could not be merged into a single network without violating this constraint, thereby identifying non-compatible groups of sequences. Conversely, while phylogenetic analysis struggled to resolve the relationships among highly similar sequences that VirNA could manage effectively, it provided a broader perspective on evolutionary connections between distinct groups of sequences. The phylogenetic analysis proved valuable in reconstructing elements of evolutionary history, especially when dealing with more divergent genomic sequences. Together, these methods offer complementary insights into the evolutionary dynamics of viral genomes.

**Figure 5:**
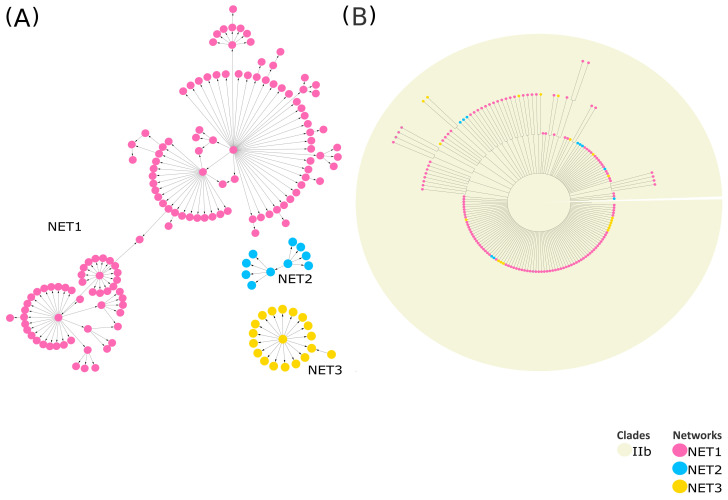
MSNs computed by VirNA on IIb MonkeyPox sequences (**A**). The phylogenetic tree is computed on the same sequences through RAxML [7] (**B**). The orientation of the arrows in the Minimum Spanning Network is based on the haplotype (from the pattern with less mutations to the pattern with more mutations). The color code of the MSNs highlights the networks identified for each Monkeypox group of non-compatible sequences by VirNA, with each network characterized by its own color. The nodes of the phylogenetic trees respect the same color code. The background of the phylogenetic tree indicates the viral variants the sequences belong to.

Part of the Monkeypox case study is presented as an example to illustrate these concepts more clearly. Comprehensive details on both the Monkeypox and SARS-CoV-2 case studies are provided in the Supplementary Materials. VirNA clusters Monkeypox sequences into three distinct, isolated networks (Figure 5, panel A), with node colors consistent across the MSNs and phylogenetic tree. The tree (Figure 5) was constructed using RAxML v8.2.12 [7] (GTRCAT model, 100 bootstrap replicates), retaining only nodes with ≥75% bootstrap support. Given the high similarity among the input sequences, the resulting tree contains numerous unresolved branches (polytomies) (Figure 5, panel B) as expected. VirNA clustering delineates three isolated subgroups, enabling a more detailed exploration of potentially significant relationships among similar haplotypes. Sequences assigned to these distinct networks by VirNA appear intermixed within the phylogenetic tree where they are reported at the same hierarchical level. Indeed, the sequences analyzed are all derived from the most recent Monkeypox outbreak of 2022 and belong to clade IIb, which accounts for the observed phylogenetic results where sequences are grouped together without distinction. On the contrary, VirNA effectively disentangles the relationships among these highly similar sequences, uncovering intriguing patterns in the resulting networks that suggest potential pathways of mutation accumulation and ancestor–descendant relationships that phylogenetic analysis struggles to unravel. Additional illustrative examples are provided in the Supplementary Materials, where phylogenetic analysis successfully reconstructs missing evolutionary relationships among more genetically divergent groups of SARS-CoV-2 sequences. These cases underscore the utility of phylogeny in revealing broader evolutionary connections between distinct and potentially diverged sequence groups, while VirNA offers a high-resolution analysis of fine-grained relationships within groups of closely related sequences.

### 2.4. Performance Test of VirNA

A performance test was carried out on a server equipped with a 512 GB System memory and two AMD EPYC 32-Core Processors. VirNA was compared with PopArt [3] and Pegas [2], focusing on computational time. Five sets of SARS-CoV-2 sequences were tested, described in the Supplementary Materials and available on the GitHub page of VirNA. VirNA is always faster than the other two tools, as shown in Supplementary Table S1 and is the only tool able to manage large datasets containing over 10,000 sequences.

## 3. Materials and Methods

### 3.1. VirNA Algorithm Details

**Figure 6:**
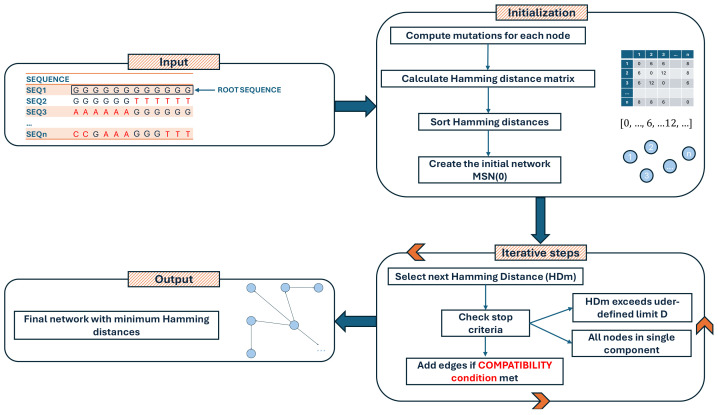
Flowchart illustrating the computational steps of the VirNA algorithm, detailing the sequential processes involved in data input, analysis, and output generation.

The software is implemented in Python (v. 3.11.4) [8] and Cython (v. 3.0.3) [9], exploiting the Numpy (v. 1.25.1) [10] library to speed up the calculation of the Hamming distance matrix, while igraph (v. 0.10.8) [11] is used for an efficient construction of the MSN. A Graph Modeling Language (GML) file is written and can be easily imported into Cytoscape (v. 3.10.2) [12], Gephi (v. 0.10) [13], or other similar tools for visualization or downstream analyses. VirNA requires a multiple alignment in FASTA format as an input file. VirNA algorithm details and definitions are reported below and are graphically described in Figure 6.

#### 3.1.1. VirNA Algorithm Definitions

- Root sequence: the first sequence of the input multiple alignment is designated as the root sequence R and will not be included in the output network. This is the reference sequence against which mutations in the dataset are identified;
- Input genomic sequences: let S = {s1, s2, …, sn} be the set of input genomic sequences;
- Mutation sets: for each sequence si, where i = 1, 2, …, n, we define a set of mutations Mj = {m1, m2, …, mp}, representing the p single-character differences (i.e., mutations) between the root sequence R and the sequence si;
- Network nodes: identical sequences are grouped in a single node. Let Gk = {s1, s2, …, sm}, with m ≤ n, be the sets of nodes in the network, where s1 = s2 = … = sm.
- Compatibility: The mutation set Mi is said to be compatible with the mutation set Mj if and only if Mi⊂Mj or Mj⊂Mi;
- Connected component: a connected component of a directed graph is defined as a subgraph where for each pair of nodes, Gi, Gj there either is a path from Gi to Gj or from Gj to Gi.

#### 3.1.2. Initialization

- For each node Gi, compute the corresponding set of mutations Mi compared to the root R;
- Calculate the Hamming distance among all sequences and store it in a list HD, HD = [HD1, 2, HD1, 3, …, HDn-1, n] where HDi, j is the Hamming distance between nodes Gi and Gj, with i: 1, …, n, j: i, …, n and i ≠ j;
- Sort the list of Hamming distances HD in ascending order removing the duplicated values: SHD = {HD1, …, HDk} where HDi in HD, k <= n(n − 1), HDi ≠ HDj for each I ≠ j, HDa < HDb for each a, b = 1, …, k if a < b;
- Create a starting network, MSN(0), where all the (grouped) sequences (nodes) are not connected.

#### 3.1.3. Iterative Steps

At each step m >= 1, let HD_m_ be the m-th distance in the sorted Hamming distances (SDH) list defined above. If no stop criteria are met (see below) the network MSN(m) is built from MSN(m − 1) by adding all the edges from Gi to Gj if and only if all the conditions below are met:

- HDi, j == HDm;
- Gi is compatible with Gj;
- Gi and Gj do not belong to the same connected component;
- Stop criteria at step m;
- All the nodes Gi 1 <= I <= n, are part of a single connected component of the current network MSN(m − 1);
- HDm > D, where HDm is the m-th distance in SDH, and D is a user-defined maximum allowable distance.

#### 3.1.4. Output

The final network represents the Minimum Hamming Distances among all input sequences.

### 3.2. Real Data, State of the Art Tools and Phylogeny

We applied VirNA, PoPArt [3], and Pegas [2], along with Maximum Likelihood and Maximum Parsimony phylogenetic algorithms, to two real sequence datasets from the GISAID repository [5], comprising SARS-CoV-2 and Monkeypox sequences, to compare the outputs of these algorithmic approaches. Details on the datasets, as well as the phylogenetic tools and parameters used, are provided in the Supplementary Materials. Both the sets of sequences are available on the GitHub page of VirNA (https://github.com/font71/VirNA, accessed on 20 February 2025).

## 4. Concluding Remarks

VirNA is specifically designed for the analysis and monitoring of rapidly evolving pathogens in large, fine-grained datasets, making it a powerful tool for pathogen surveillance. A Beta version of VirNA was employed to identify viral haplotypes entering and leaving Trento and Vo’ areas (Italy) during the SARS-CoV-2 pandemic [6,14]. Unlike previous methods [4], VirNA introduces a haplotype compatibility criterion and directional connections among nodes that reflect the accumulation of mutations, ensuring that only the most closely related sequences are linked. This approach produces evolutionary plausible networks, particularly suited for fast-evolving pathogens. By applying the compatibility criterion, VirNA enables users to clearly differentiate genomically distant sequences that are co-circulating within the same time period, generating distinct networks whose evolutionary relationship can be explored through phylogenetic analysis. VirNA excels in analyzing large, complete, and well-curated datasets, making it an ideal tool for studying rapid pathogen evolution over short time scales, such as those observed during pandemic surveillance.

## Data Availability

The findings of this study are also based on SARS-CoV-2 sequences, and associated metadata are available on GISAID and accessible at https://doi.org/10.55876/gis8.240212ht, and MonkeyPox sequences and associated metadata are available on GISAID (acknowledgements table available in the Supplementary Materials).

## Supplementary Materials

The following supporting information can be downloaded at: https://www.mdpi.com/article/10.3390/ijms26052008/s1.
