# Supplementary material for: Viral Network Analyzer (VirNA): A Novel Minimum Spanning Networks Algorithm for Investigating Viral Evolution
Source: Int J Mol Sci. 2025 Feb 25;26(5):2008. doi: 10.3390/ijms26052008 (PMC11900457; doi:10.3390/ijms26052008)
Supplement: Supplementary file 1 [file ijms-26-02008-s001.zip › Supplementary_files/gisaid_supplemental_table_epi_set_240212ht_SARS.pdf]

SUPPLEMENTAL TABLE

Data Availability

GISAID Identifier:	EPI_SET_240212ht
doi:	10.55876/gis8.240212ht

All genome sequences and associated metadata in this dataset are published in GISAID's EpiCoV database. To view the contributors of each individual sequence with details such as accession number, Virus name, Collection date, Originating Lab and Submitting Lab and the list of Authors, visit 10.55876/gis8.240212ht

Data Snapshot

- EPI_SET_240212ht is composed of 406,219 individual genome sequences.
- The collection dates range from 2020-02-27 to 2022-05-14;
- Data were collected in 4 countries and territories;
- All sequences in this dataset are compared relative to hCoV-19/Wuhan/WIV04/2019 (WIV04), the official reference sequence employed by GISAID (EPI_ISL_402124). Learn more at https://gisaid.org/WIV04.
